# Four dimensional-scanning transmission electron microscopy study on relationship between crystallographic orientation and spontaneous polarization in epitaxial BiFeO_3_

**DOI:** 10.1038/s41598-024-66382-6

**Published:** 2024-07-05

**Authors:** In-Tae Bae, Brendan Foran, Hanjong Paik

**Affiliations:** 1 Microeletronics Technology Department, The Aerospace Corporation, El Segundo, CA 90245 USA; 2 School of Electrical and Computer Engineering, University of Oklahoma, Norman, OK 73019 USA; 3 Center for Quantum Research and Technology, University of Oklahoma, Norman, OK 73019 USA

**Keywords:** Materials science, Condensed-matter physics, Materials for devices

## Abstract

Spontaneous polarization and crystallographic orientations within ferroelectric domains are investigated using an epitaxially grown BiFeO_3_ thin film under bi-axial tensile strain. Four dimensional-scanning transmission electron microscopy (4D-STEM) and atomic resolution STEM techniques revealed that the tensile strain applied is not enough to cause breakdown of equilibrium BiFeO_3_ symmetry (rhombohedral with space group: *R3c*). 4D-STEM data exhibit two types of BiFeO_3_ ferroelectric domains: one with projected polarization vector possessing out-of-plane component only, and the other with that consisting of both in-plane and out-of-plane components. For domains with only out-of-plane polarization, convergent beam electron diffraction (CBED) patterns exhibit “extra” Bragg’s reflections (compared to CBED of cubic-perovskite) that indicate rhombohedral symmetry. In addition, beam damage effects on ferroelectric property measurements were investigated by systematically changing electron energy from 60 to 300 keV.

## Introduction

BiFeO_3_ (BFO) is the only known material that shows two primary ferroic orders, i.e., ferroelectricity and antiferromagnetism, simultaneously above room temperature^1,2^. It has been intensively studied for a couple of decades to test the feasibility of electric field control of magnetism, which implies ground-breaking potential for electronics applications such as multiply controlled devices^3–5^. The primary method used to grow BFO is epitaxy via pulsed laser deposition, molecular beam epitaxy, or ultra-high vacuum sputtering. The technological advantage of epitaxial growth is to allow for modification of BFO characteristics by imparting elastic strain (caused by lattice mismatch with substrate material) without causing lattice defects such as misfit dislocations ^6^. Since crystal symmetry information is closely associated with properties, such as spontaneous polarization, understanding of the relationships between crystal symmetry, orientation and spontaneous polarization is of great technological importance.

A variety of metastable BFO phases have been identified, experimentally and theoretically, as dependent on: (1) the amount of elastic strain and (2) type of elastic strains, i.e., either compressive or tensile. It is worth noting that the magnitudes of spontaneous polarization reported for metastable BFO phases found experimentally were similar to that of equilibrium BFO (i.e., 90–100 μC/cm^2^). This is in disagreement with higher values predicted by theoretical calculations^7,8^.

While some of the metastable BFO phases are reported with complete symmetry information such as space group, lattice parameter, and basis atom locations, others are simply based on lattice parameter distortions measured by X-ray diffraction and include no such information^7–18^. Most of the previously reported metastable BFO phases have used a pseudocubic (*pc*) notation for equilibrium BFO with a lattice parameter of ~ 0.396 nm to evaluate the elastic strain. However, the *pc* notation of equilibrium BFO disregards two crucial rhombohedral characteristics, i.e., (1) ~ 0.5° rhombohedral distortion and (2) basis atom location shifts caused by *oxygen octahedron rotation* in equilibrium BFO; thus the *pc* approximation does not represent *true* symmetry in equilibrium BFO^19–21^.

Most of the experimentally found metastable BFO phase identifications were made based on small distortions relative to *pc* notation-approximated equilibrium BFO unit cell. Few of these studies have shown electron diffraction patterns from metastable BFO phases with appropriate detailed analysis to effectively evaluate symmetry utilizing unit cell distortions with accurate *basis atom* positions. For example, nano-beam electron diffraction patterns combined with structure factor calculation (that makes use of *true* equilibrium BFO symmetry using *hexagonal* notation) unambiguously demonstrated the existence of Bragg’s reflections at *Q* (scattering vector) ≈ 4.18 nm^−1^ specifically tied to a rhombohedral distortion, i.e., oxygen octahedral rotation, in equilibrium BFO phase^21^. While the Bragg’s reflections can be readily used to distinguish rhombohedral BFO from other metastable BFO phases (owing to their exclusive association with rhombohedral BFO), only a few studies implementing the Bragg’s reflections have been reported^22–25^. Thus, the relationship between the crystal symmetries of metastable BFO phases and their spontaneous polarization property remain debated.

In this study, four dimensional-scanning transmission electron microscopy (4D-STEM) technique^26,27^ is applied to an epitaxial BFO film designed to be under tensile strain using PrScO_3_ (PSO) single crystal to investigate: (1) crystal symmetry within BFO film, (2) ferroelectric domain structure within BFO film in terms of the relationship between spontaneous polarization and crystallographic orientations, and (3) beam-damage effects on measured ferroelectric domain structure.

## Results and discussion

**Figure 1:**
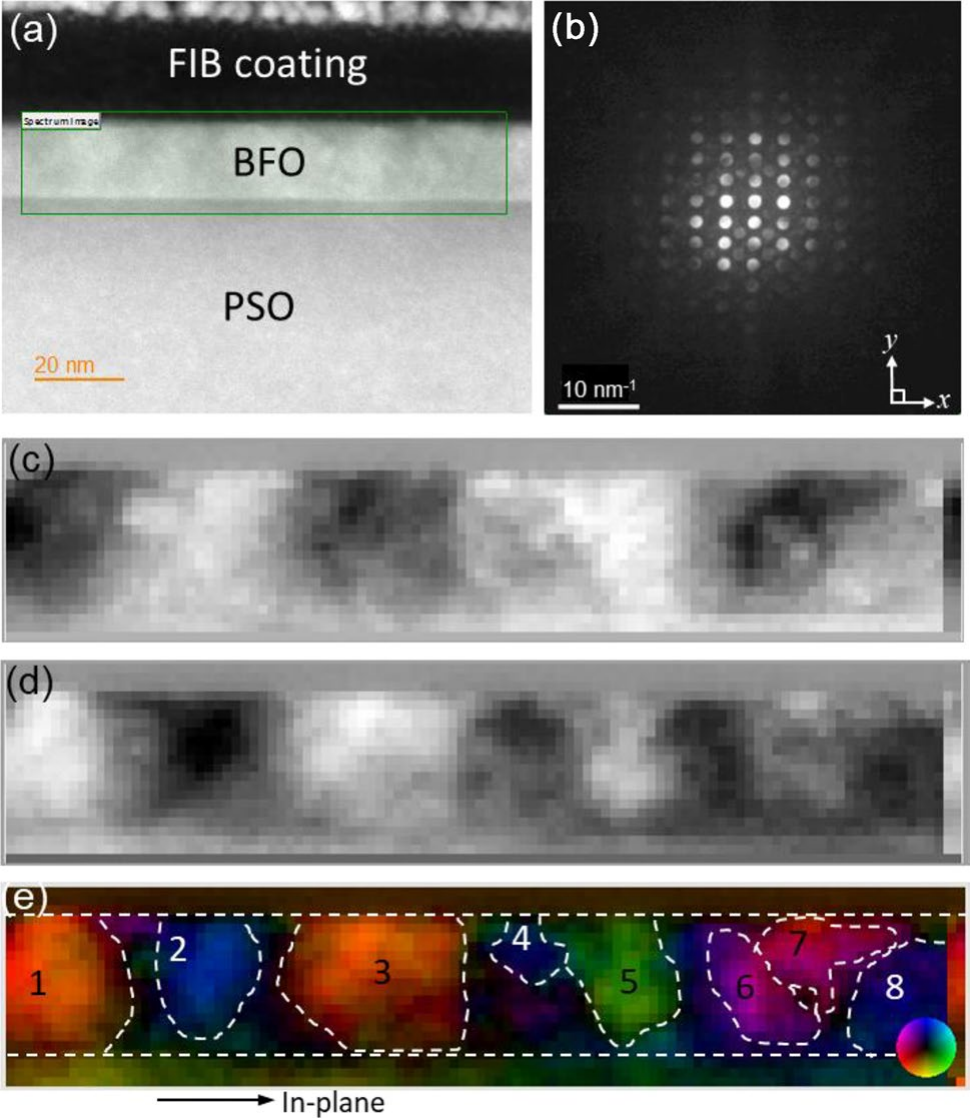
(**a**) A cross-sectional HAADF-STEM image of epitaxial BFO grown on PSO along $[\overline{1 }11]$_o_ zone axis using 120 keV electron probe (**b**) an example of CBED pattern from BFO, (**c**) the measured shift in zeroth order diffraction disks in CBED patterns along *x* direction, i.e., *dx*, (**d**) the measured shift in zeroth order diffraction disks in CBED patterns along *y* direction, i.e., *dy*, (**e**) vector displacement map with a color wheel as an inset bottom right corner. Ferroelectric domains are denoted by white-dashed lines with numbers.

Figure 1a shows a high angle annular dark field (HAADF)-STEM image of the BFO films grown on (101)_o_ PSO substrate (space group: *Pnma*, *a* = 0.5780 nm, *b* = 0.8025 nm, *c* = 0.5608 nm, *α* = *β* = *γ* = 90°) along $\left[ {\overline{1}11} \right]$_o_ (subscript “o” stands for orthorhombic notation) zone axis using 120 keV electron probe^28^. ~ 20 nm BFO epitaxial film shows up brighter than underlying PSO substrate because Bi atom within BFO, which is heavier than Pr and Sc in PSO, provides more signals to the HAADF detector located at the collection semi-angle of 80–100 mrad^29^. A green rectangle in Fig. 1a indicates the area where a 4D-STEM data set, two probe-scanning dimensions in real space and two momentum dimensions in reciprocal space, were acquired using 120 keV electron probe as shown in Fig. 1b–e. Figure 1b shows an example of a convergent beam electron diffraction (CBED) pattern that was collected from each scanning position in Fig. 1c–e. While 4D-STEM data acquisition with sub-angstrom aberration corrected electron probes is known to be advantageous to visualize the potential gradient across single atomic columns and the nuclear charge in GaN and SrTiO_3_^30–32^, sub-angstrom electron probes with large convergence angles cause Bragg’s reflections in CBED patterns to overlap, which complicates measurement of long range electric fields arising from ionicity because 4D-STEM signal is dominated by nuclear potential^33,34^. Thus, a small convergence semi-angle of ~ 1.26 mrad is used to prevent Bragg’s reflection overlaps as shown in Fig. 1b. The spontaneous polarization orientations within BFO domains were determined by analysis of zeroth order CBED pattern shift along two orthogonal directions, i.e., *x* and *y*, as denoted in Fig. 1b. These shifts are known to occur due to the deflection of the incident electron beam by average electric field over unit cell in ferroelectric materials, whereas asymmetric intensities in conjugate disks resulting from Friedel’s law breakage allows for polarity field measurement^26,27,33,34^. While no obvious contrast resulting from spontaneous polarization is seen from BFO layer in HAADF-STEM image (see Fig. 1a), areas with distinctively bright and dark contrasts are seen in Fig. 1c and d. The color-coded vector displacement map, based on Fig. 1c and d, is shown in Fig. 1e. Note that the intensity scales to the magnitude of the vector field and the color represents its orientation as shown by the color wheel at bottom-right corner. Ferroelectric domains with sizes ranging from ~ 10 to ~ 25 nm are clearly identified. Table 1 summarizes the distribution of spontaneous polarization orientations in each domain with respect to the in-plane orientation denoted in Fig. 1e. Note that the polarization angles measured are based on projection along $\left[ {\overline{1}11} \right]$_o_ PSO orientation. The mean and standard deviation are based on 10 pixels from the central area of each ferroelectric domain. It is worth noting that domains 2, 4, and 8 possess only an out-of-plane component of polarization with polarization angles ~ − 90°. All other domains have both of out-of-plane and in-plane polarization components, i.e., their polarization angles ≠  ± 90°.

**Table 1:** Summary of the spontaneous polarization angles, i.e., mean and standard deviation, with respect to the *in-plane* orientation denoted as an arrow in Fig. 1e.

Domain	Angle with respect to in-plane orientation (deg.)	
	Mean	Standard deviation
1	135.2	3.5
2	− 87.7	4.8
3	133.4	3.4
4	− 90.2	4.8
5	46.7	3.8
6	− 154.9	6.5
7	155.2	9.1
8	− 90.5	3.8

**Figure 2:**
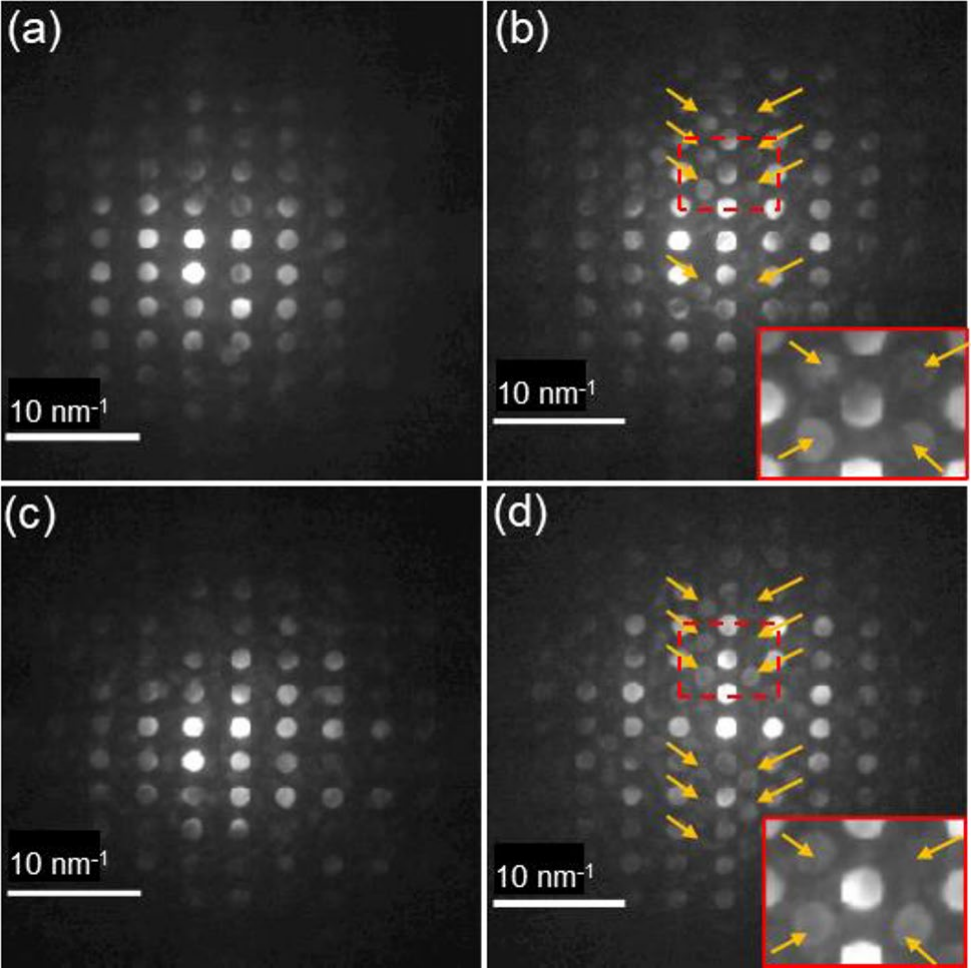
CBED patterns acquired from domains 1 (**a**), 2 (**b**), 3 (**c**), and 4 (**d**) using 120 keV electron probe. Extra Bragg’s reflections are denoted by orange arrows in (**b**) and (**d**). The two boxed areas in red in (**b**) and (**d**) are magnified as insets at the bottom-right corner, respectively.

Prior to further discussion on the relationship between spontaneous polarization and crystallographic orientations, crystal symmetry within the BFO films needs to be identified. CBED patterns from domains 1 through 4, extracted from 4D-STEM data set, are shown in Fig. 2. While Fig. 2a–d all exhibit fundamental Bragg’s reflections, extra Bragg’s reflections (denoted by orange arrows) can be observed in Fig. 2b and d only. The two boxed areas in red in Fig. 2b and d are magnified as insets at the bottom-right corner, respectively. This clearly indicates that while Fig. 2a and c correspond to [110]_h_ (subscript “h” denotes three index hexagonal notation) zone axes of rhombohedral BFO, i.e., equilibrium BFO, Fig. 2b and d match [$\overline{1}11]$_h_ zone axis of rhombohedral BFO (see Table S1 for *h* to *pc* notation conversion). This result is in good agreement with a recent work that performed nano-beam electron diffraction analysis for BFO grown on PSO substrate^21^. Note that [110]_h_ and [$\overline{1}11]$_h_ zone axes found in the present work are crystallographically equivalent to [$0\overline{1}0$]_h_ and [211]_h_ zone axes found in the recent work^21^, respectively, as the angles between the corresponding orientations are 120° and the CBED patterns of the corresponding orientations are identical (see Figure S1). In addition, the work showed that the extra Bragg’s reflections shown in Fig. 2b and d are the result of oxygen octahedral rotation occurring in rhombohedral BFO (space group: *R3c*; lattice parameter: *a* = 0.5678 nm, and *c* = 1.3982 nm) by demonstrating that the electron diffraction simulation of *pc* notation-approximated BFO (space group: $Pm\overline{3}m$; lattice parameter *a* = 0.396 nm) that possesses no oxygen octahedral rotation exhibits no such extra Bragg’s reflections^21^.

**Figure 3:**
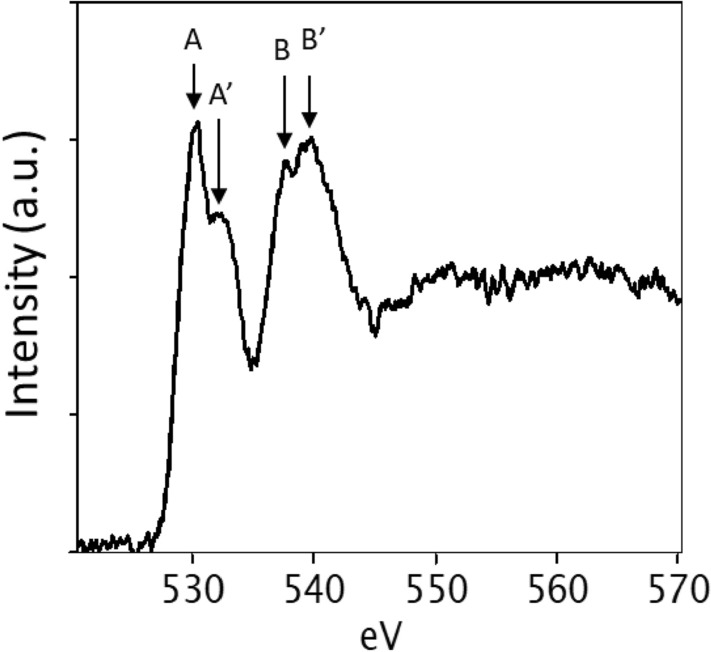
An EELS on O *K*-edge from BFO film using 120 keV electron probe with ~ 1.0 eV energy resolution.

Since local electronic structure in O (oxygen) *K*-edge in BFO is known to be sensitive to local bonding and geometry^18^, an electron energy loss spectrum (EELS) is acquired from the BFO film with ~ 1.0 eV FWHM energy resolution (see Fig. 3). The O *K*-edge spectral features can be discussed for two regions, as defined by the labels A, A’ and B, B’ as shown in Fig. 3. Peaks A and A’ are readily attributed to hybridization between O 2*p* and Fe 3*d* states and a transition between O 2*p* and Bi 5*d* (or 6*d*) states, respectively^35–37^.peaks B and B’ are associated with hybridization between O 2*p* and Fe 4*sp* states in bulk and thin BFO^35,36,38^. The relative intensities of A and A’, and of B and B’ are in good agreement with those for bulk and thin film rhombohedral BFO, where the bonding geometry between Fe and O atoms is octahedral^23,35–37^. Note that these are in disagreement with metastable BFO phases that show the relative intensity inversion for B and B’^17,18,36^. Accordingly, the EELS result on O K-edge film is consistent with the rhombohedral symmetry found from CBED patterns in Fig. 2.

**Figure 4:**
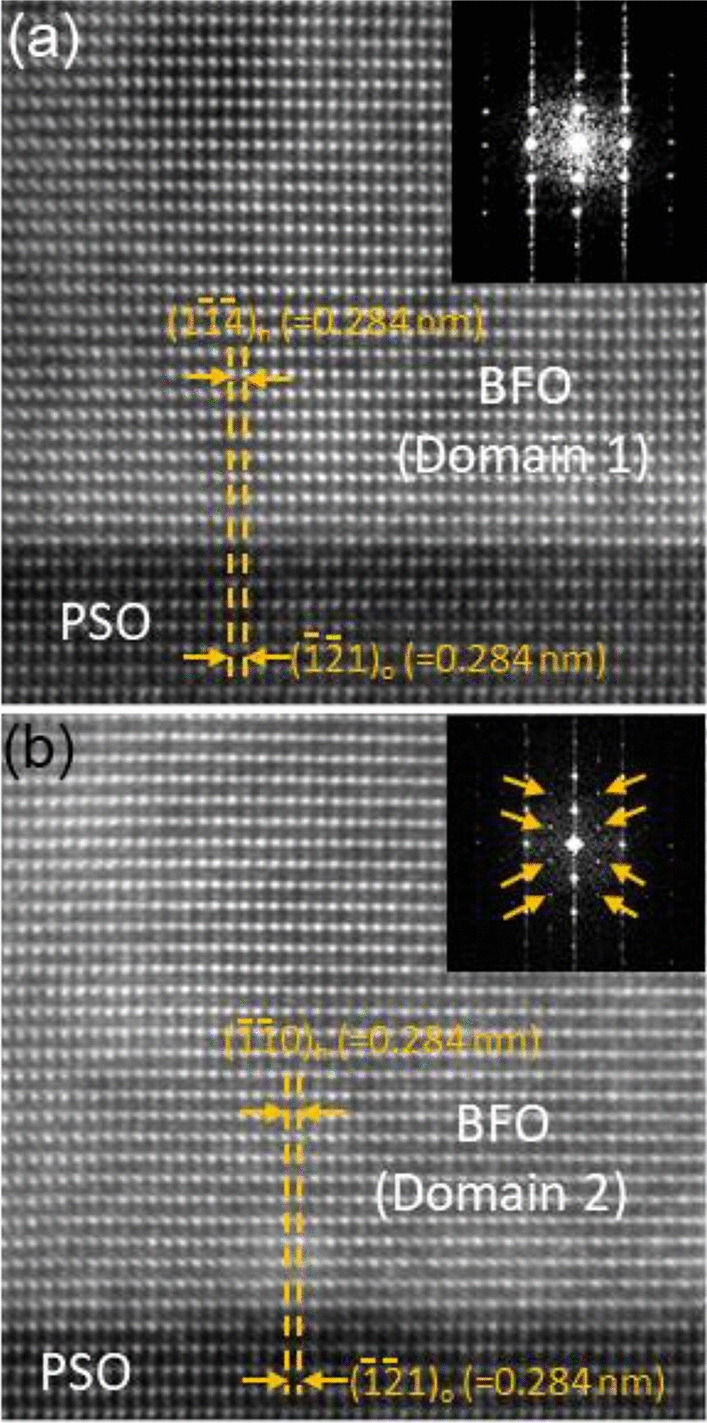
(**a**) Atomic resolution HAADF-STEM images from (**a**) BFO domain 1/PSO and (**b**) BFO domain 2/PSO interfaces along PSO $[\overline{1 }11]$_o_ zone axis. The interplanar distances of BFO I and BFO II along in-plane orientation are the same as those of PSO with no sign of misfit dislocations at the interfaces. FFT patterns from domains 1 and 2 are shown as insets at the top-right corner in (**a**) and (**b**), respectively, with extra Bragg’s reflections from domain 2 denoted with orange arrows.

Figure 4 shows two atomic resolution HAADF-STEM images for the BFO/PSO interface [recorded at domains 1 and 2, respectively, of Fig. 1e] to investigate strain within BFO film which is expected from lattice misfit (~ 1.5%) with PSO^6,21,39^. The PSO lattice spacing along the in-plane orientation, i.e., ($\overline{12} 1$)_o_, is commensurate with that of the BFO, i.e., $\left( {1\overline{14} } \right)$_h_, in domain 1 (see Fig. 4a) and ($\overline{11} 0$)_h_ in domain 2 (see Fig. 4b), with no sign of misfit dislocations despite ~ 1.5% of lattice misfit. Since misfit dislocations, known to relax elastic strain when the density is higher than a threshold value, are not found, the elastic strain resulting from ~ 1.5% of lattice misfit with PSO is thought to be maintained within BFO film. Fast Fourier transform (FFT) patterns from domains 1 and 2 (see insets in Fig. 4a and b) show the same characteristics as found in CBED patterns, i.e., extra Bragg’s reflections (denoted by orange arrows in the inset of Fig. 4b) were found for domain 2 only. Thus, the result in FFT analysis of atomic resolution HAADF-STEM data is consistent with that of CBED patterns.

The combined evidence of (1) rhombohedral symmetry (shown in Figs. 1, 2 and 3) and (2) ~ 1.5% tensile elastic strain (shown in Fig. 4), suggests that this level of strain falls below the threshold for equilibrium symmetry breakdown in this epitaxial BFO film. This result agrees with prior structural flexibility in rhombohedral BFO discussed previously in terms of : (1) a small perovskite tolerance factor (~ 0.88) allowing for large degree of rotation and/or tilting of oxygen octahedra^40^, (2) variation in experimentally found lattice parameters for bulk rhombohedral BFO (i.e., ~ 0.82% in *a* and ~ 0.71% in *c* in hexagonal notation^41^), and (3) availability of multiple meta stable phases^7,8^.

**Figure 5:**
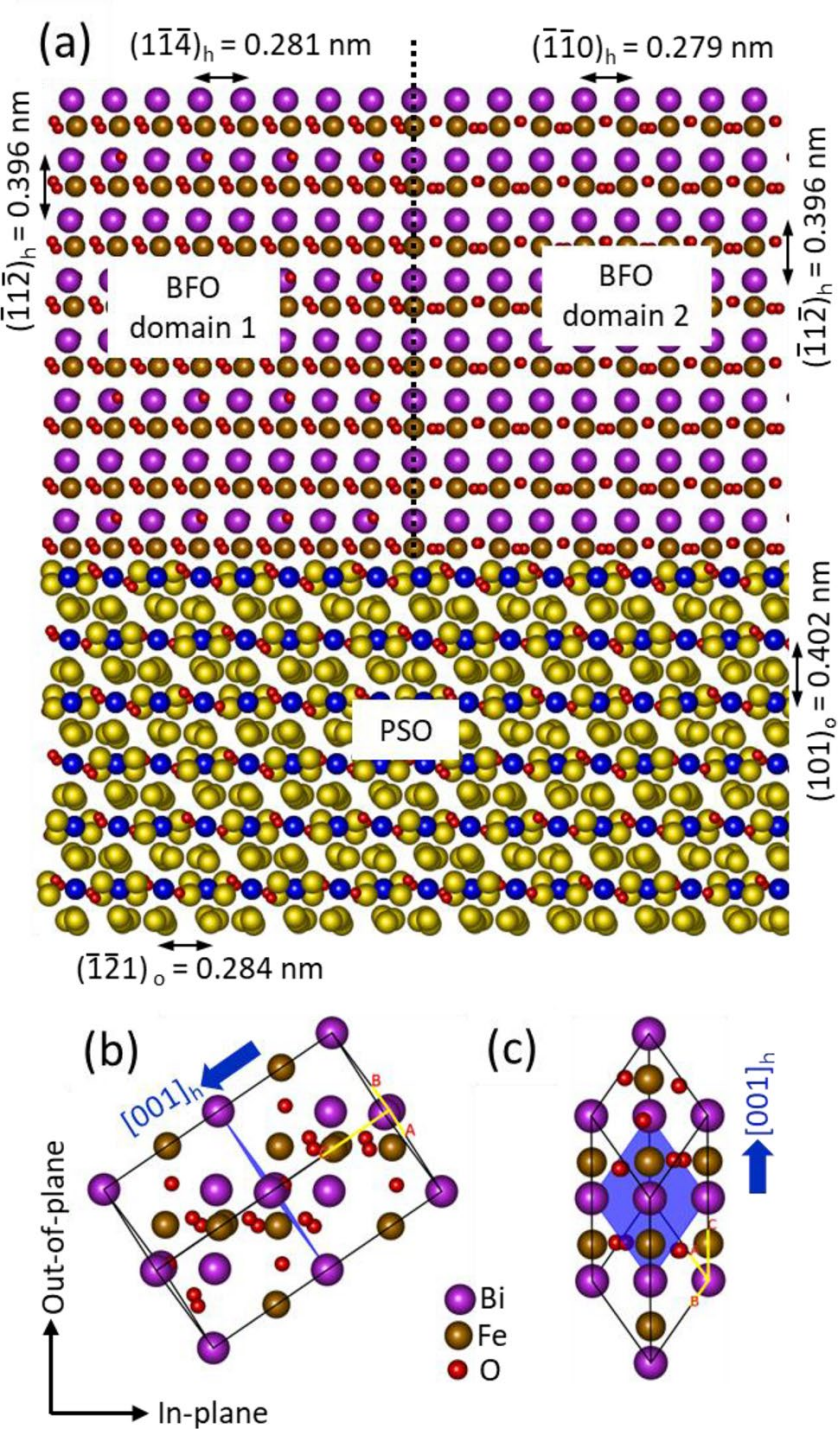
(**a**) Atomic model showing the epitaxial relationship between BFO domains 1 and 2 with respect to PSO substrate. Note that the out-of-plane interplanar distances between the two domains are identical. Spontaneous polarization orientations in BFO domains 1 (**b**) and 2 (**c**) are shown with blue arrows. Note that the BFO unit cell is projected along the corresponding zone axes of each BFO domain, i.e., [110]_h_ for domain 1 and [$\overline{1 }11]$_h_ for domain 2. (001)_h_ plane in each BFO domain is denoted in blue with the BFO unit cell.

Let us turn our attention to the relationship between the spontaneous polarization orientations found in ferroelectric domains and their crystallographic orientations. Based on the results found in Figs. 1 and 2, an atomic model is constructed as shown in Fig. 5. Note that *no tensile strain* is assumed within BFO ferroelectric domains. Figure 5a shows that the *unstrained* interplanar distances between domains 1 and 2 are the same along out-of-plane [see ($\overline{1}1\overline{2}$)_h_ in domains 1 and 2] indicating this interface [see dotted line in Fig. 5a] shows no misfit strain. Note that the HAADF-STEM image showed no distinctive contrast across this interface because of: (1) no misfit stain field and (2) the close relationship of the projected crystal structures in these two different orientations. The differences in these crystal orientations are subtle and are not well distinguished when a *pc* notation is used to describe the equilibrium BFO lattice. Figure 5b and c show the rhombohedral BFO unit cell in hexagonal notation with zone axes aligned along [110]_h_ (for domain 1) and [$\overline{1}11]$_h_ (for domain 2), respectively. The spontaneous polarization orientations, i.e., [001]_h_ orientation (= [111]_*pc*_), are denoted with the arrows in blue. The basal plane, i.e., (001)_h_ plane [= (111)_*pc*_ plane], is also denoted in blue in each figure. Note that this plane in Fig. 5b is near perpendicular to the plane of the page and thus is almost hidden. The spontaneous polarization angles are calculated to be ~ 144° for domain 1 and ~ − 90° for domain 2 with respect to in-plane orientation. While the ~ 144° value for domain 1 is ~ 9° from ~ 135.2° measured by 4D-STEM, the ~ -90° value for domain 2 matches more closely to the ~ − 87.7° measured by 4D-STEM (see Table 1). Elastic strain in epitaxially grown BFO is known to cause spontaneous polarization orientation to change within (110)_*pc*_ plane from the calculated angles of ~ 144° and ~ − 90° based on *unstrained* BFO^39,42^. Besides, polarity effect (showing up with asymmetric intensities in conjugate disks resulting from Friedel’s law breakage) could contribute the spontaneous polarization orientation change. Note that (110)_*pc*_ plane in Fig. 5b, i.e., domain 1, is parallel to the plane of the page whereas that in Fig. 5c, i.e., domain 2, is perpendicular to the plane of page. Thus, the polarization rotation caused by ~ 1.5% tensile strain is more readily measured in domain 1 than in domain 2. Based on the results in Figs. 1, 2 and 5, three dimensional polarization vectors for domains 1 and 2 are schematically drawn in Figure S2. Both of polarization vectors run along either of PSO diagonal orientations with 45.2 ± 3.5° off the BFO film surface in domain 1 (see Fig. S2a) and 35.6 ± dev° off the BFO film surface in domain 2 (see Fig. S2b). Note that while 45.2 ± 3.5° in domain 1 is based on 4D-STEM measurement, 35.6 ± dev° is an assumption defined by the sum of theoretical value of 35.6° for unstrained BFO^43^ and (2) polarization vector change (dev.) owing to the tensile strain caused by PSO substrate. A recent aberration corrected TEM work on an epitaxial BFO film grown on PSO substrate revealed that while 70° ferroelectric domain wall consists of polarization components of both in-plane and out-of-plane orientations, 180° domain wall is comprised of out-of-plane polarization component only^44^. Note that 70° and 180° represent the angles between two polarization vectors across domains. Since the projection orientation of BFO ferroelectric domain in the report is along [110]_*pc*_ orientation, which is equivalent to [110]_h_ (for domain 1) and [$\overline{1}11]$_h_ (for domain 2) in the current work, the conclusion in the prior report is consistent with the current one based on Figs. 1, 2 and 5.

Note that while the CBED pattern from domain 1 shows no extra Bragg’s reflections (see Fig. 1a), that from domain 2 exhibits the extra Bragg’s reflection (see Fig. 1b). Thus, the ferroelectric domain with no extra Bragg’s reflections has spontaneous polarization orientation with both in-plane and out-of-plane components, whereas the one exhibiting extra Bragg’s reflections possesses only an out-of-plane polarization component. This indicates that polarization orientation within BFO ferroelectric domains can be identified by the existence of extra Bragg’s reflections in CBED pattern from BFO ferroelectric domain.

**Figure 6:**
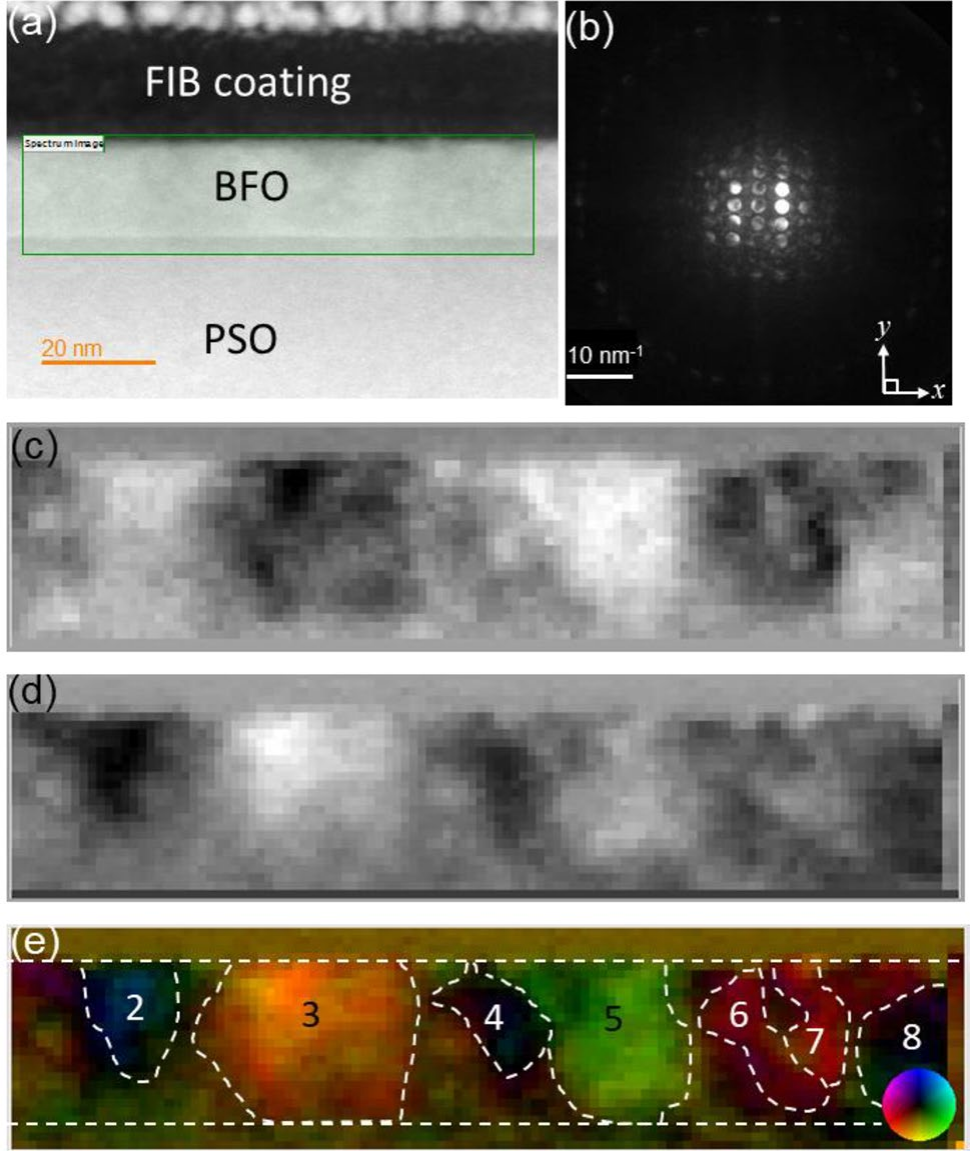
(**a**) A cross-sectional HAADF-STEM image of epitaxial BFO grown on PSO along $[\overline{1 }11]$_o_ zone axis using 60 keV electron probe, (**b**) an example of CBED pattern from BFO, (**c**) the measured shift in zeroth order diffraction disks in CBED patterns along *x* direction, i.e., *dx*, (**d**) the measured shift in zeroth order diffraction disks in CBED patterns along *y* direction, i.e., *dy*, (**e**) Vector displacement map with a color wheel as an inset bottom right corner. Ferroelectric domains are denoted by white-dashed lines with numbers.

**Figure 7:**
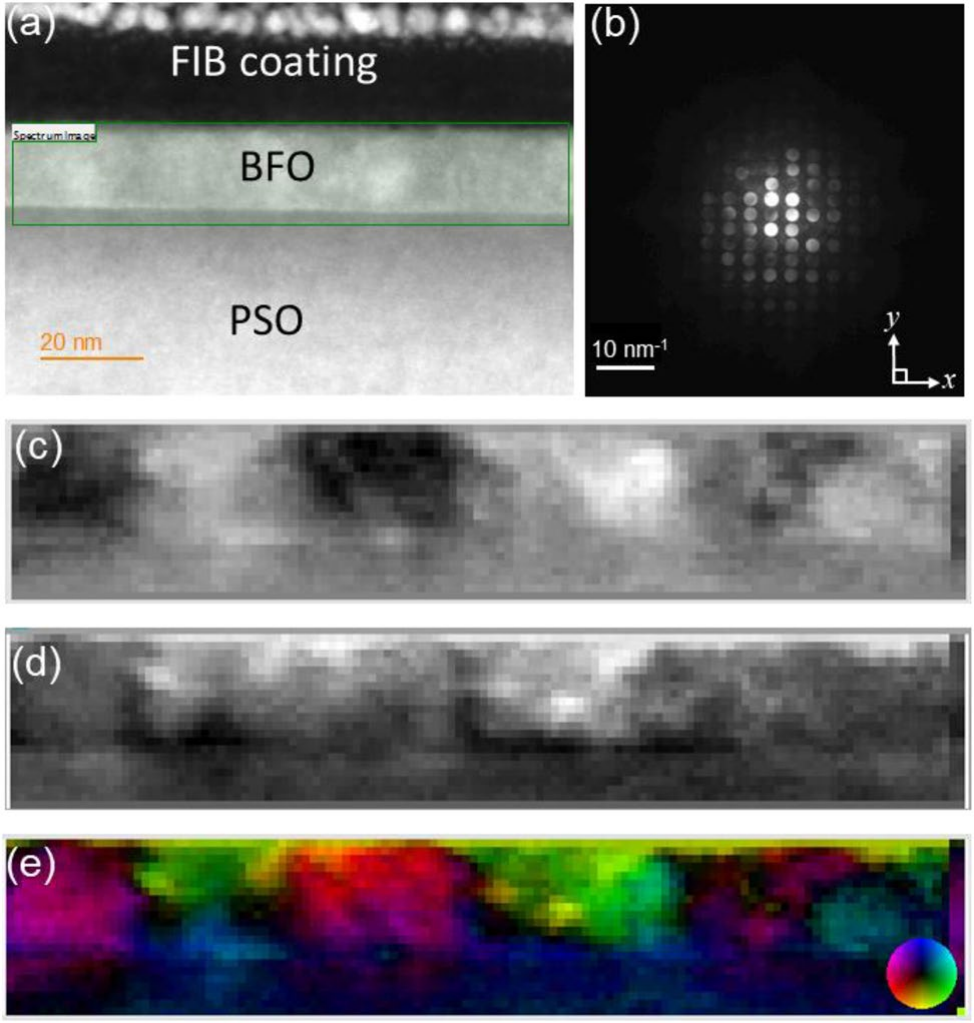
(**a**) A cross-sectional HAADF-STEM image of epitaxial BFO grown on PSO along $[\overline{1 }11]$_o_ zone axis using 300 keV electron probe, (**b**) an example of CBED pattern from BFO, (**c**) the measured shift in zeroth order diffraction disks in CBED patterns along *x* direction, i.e., *dx*, (**d**) the measured shift in zeroth order diffraction disks in CBED patterns along *y* direction, i.e., *dy*, (**e**) Vector displacement map with a color wheel as an inset bottom right corner.

To investigate probe-beam damage effect on ferroelectric domain, 4D-STEM data were acquired at 60 and 300 keV as shown in Figs. 6 and 7. Figures 6a and 7a are the HAADF images, acquired from the *same* area as in Fig. 1a, at each keV, showing no sign of contrast that could be associated with ferroelectric domains. The examples of CBED pattern obtained at each keV are shown in Figs. 6b and 7b. Note that Bragg’s reflections in the CBED patterns separate related to convergence angle adjustment (i.e., ~ 5.25 mrad for 60 keV and ~ 1.84 mrad for 300 keV). The color-coded vector displacement maps at each keV, based on shifts in zeroth order diffraction disks in CBED patterns (see Fig. 6c, d for 60 keV and Fig. 7c and d for 300 keV) are shown in Figs. 6e and 7e. Note that the intensity scales to the magnitude of the vector field and the color represents its orientation as shown by the color wheel at bottom-right corner in each figure. It can be readily noticed that while the shapes and colors of ferroelectric domains in Fig. 6e are comparable to those in Fig. 1e, those of ferroelectric domains in Fig. 7e are different than those in Fig. 1e in terms of domain morphology and colors, i.e., polarization orientations. In particular, the bottom quarter of BFO layer shows low signal/noise (S/N) ratio in Fig. 7e indicating that while 120 and 60 keV probe energies induced no noticeable beam-damage, the 300 keV probe energy damaged BFO ferroelectric domain ordering. It is well known that high energy electron beams may cause both ionization and displacement damages. Since ionization damage decreases with increasing electron acceleration voltage^45,46^, the beam damage found at 300 keV is most likely attributed to displacement damage. When incident electron provides recoil energy greater than threshold displacement energy, *E*_*d*_, of each constituent atoms within target material, point defects, such as Frenkel pairs are introduced by knocked-on atoms. The maximum recoil energy, $T_{m}$, that an incident electron transfers to constituent atoms in target material is given by^46^:

$T_{m} = 2E\left( {E + 2m_{0} c^{2} } \right)/Mc^{2}$,where E is the energy of incident electron, $m_{0}$ the rest mass of electron, $c$ the velocity of light, and $M$ the mass of the displaced atom. Although *E*_*d*_ necessary to create knock-on damage and make stable point defects (such as Frenkel pair) have not yet been determined for BFO, ~ 25 eV was proposed as a general guideline of threshold displacement energy^47^. Calculated $T_{m}$ for Bi, Fe and O atoms at 60, 120, and 300 keV are summarized in Table 2. While most of the $T_{m}$ values are less than ~ 25 eV of the suggested *E*_*d*_, the $T_{m}$ of ~ 53.2 eV found for O atoms at 300 keV is significantly greater than the suggested *E*_*d*_ indicating that 300 keV electrons likely cause displacement damage in BFO through accumulation of O vacancies and interstitials. Previous theoretical studies discussed that O-poor conditions provide fully ionized oxygen vacancies which pair with cation atom to lead to local ferroelectric polarization called imprint effect which disturb spontaneous polarization within BFO^48,49^.

**Table 2:** Summary of maximum recoil energy, *T*_*m*_, for Bi, Fe, and O atoms against electron probe energy ranging from 60 to 300 keV.

Electron energy (keV)	Maximum recoil energy, *T*_*m*_, (eV)		
	Bi	Fe	O
60	~ 0.7	~ 2.5	~ 8.7
120	~ 1.4	~ 5.3	~ 18.4
300	~ 4.1	~ 15.2	~ 53.2

Since electron beam capable of providing $T_{m}$ that is greater than *E*_*d*_ to constituent atoms is known to knock-off the atoms from exit surface of the sample through displacement damage process^50,51^, it is reasonably assumed that 300 keV electron probe used in the current study can cause displacement damage leading to O-poor condition within BFO leading to disturb spontaneous polarization through imprint effect. This can be the reason of modified shapes and colors with low S/N ratio area found in measured ferroelectric domains by 300 keV electron probe as shown in Fig. 7e.

## Summary

4D-STEM technique was applied to an epitaxial BFO film engineered to be under ~ 1.5 % of bi-axial tensile strain using PSO single crystal substrate. Our key results include:

1. Color-coded vector displacement map derived from 4D-STEM center of mass deflection measurements identified BFO ferroelectric domains with sizes ranging from ~ 10 to ~ 25 nm. Two types of ferroelectric domains were observed, i.e., one with both in-plane and out-of-plane polarization components and the other with an out-of-plane polarization only.
2. Further comparison with CBED patterns acquired from the ferroelectric domains indicates correlation between extra Bragg’s reflections and the polarization component characteristic, i.e. extra Bragg’ reflections indicate out-of-plane polarization only; no extra Bragg’s reflection leads to both in-plane and out-of-plane polarization components within BFO ferroelectric domains.
3. While atomic resolution HAADF images indicates ~ 1.5% biaxial tensile strain within BFO film elastically, CBED and EELS analyses suggest that the strain is not enough to cause rhombohedral symmetry breakdown within BFO film.
4. Comparison of 4D-STEM data recorded at different incident electron probe energies (60, 120, and 300 keV tested) identified that displacement damage observed at 300 keV could reduce (and modify) measurable ferroelectric property within BFO film through possibly O vacancy formation.

## Methods

An epitaxial BFO film of ~ 20 nm was grown on a (101)_o_ PSO substrate using molecular beam epitaxy in PAPADIM facility at Cornell University. The cross-sectional sample preparation for 4D-STEM measurement was performed using a Ga ion Dual beam focused ion beam, Thermo Fisher Helios 600. Ga ion energy was gradually decreased from 30 to 2 kV to minimize ion beam induced damage. A Thermo Fisher Titan Themis G2 300 equipped with a probe corrector was used for 4D-STEM data acquisition at acceleration voltages ranging from 60 to 300 keV. The convergence semi-angles of electron probe were adjusted between ~ 1.25 to ~ 5.25 mrad to separate Bragg’s reflections in CBED patterns. CBED patterns were calibrated using [202] and [$\overline{2}0\overline{2}$] Bragg’s reflections of PSO substrate. Two different values of camera length, i.e., 160 (for 60 kV) and 300 (for 120 and 300 kV) mm were used to collect large spatial frequency (up to ~ 20 nm^−1^) information in CBED patterns. A Gatan OneView™ CMOS camera with readout binned to 512 × 512 pixels was used to collect diffraction data for 4D STEM. HAADF-STEM images and 4D-STEM were collected with ~ 176° image rotation with respect to CBED patterns. The Gatan Microscopy Suite software was used to analyze 4D STEM data using a center of mass method that fits shifts across the full CBED pattern at each pixel position. The image rotation of ~ 176° was compensated before center of mass data process. A Gatan Image Filter (GIF) Quantum was used to acquire EELS data.

### Supplementary Information

Supplementary Information.

## Data Availability

All data generated or analyzed during this study are included in the published article. In case, one wished to consult further information, it will be made available through the corresponding author on reasonable request.
