# Supplementary material for: Four dimensional-scanning transmission electron microscopy study on relationship between crystallographic orientation and spontaneous polarization in epitaxial BiFeO3
Source: Sci Rep. 2024 Jul 5;14:15513. doi: 10.1038/s41598-024-66382-6 (PMC11226628; doi:10.1038/s41598-024-66382-6)
Supplement: Supplementary file 1 — Supplementary Information. [file 41598_2024_66382_MOESM1_ESM.docx]

Four dimensional-scanning transmission electron microscopy study on relationship between crystallographic orientation and spontaneous polarization in epitaxial BiFeO_3_

In-Tae Bae^1,^*, Brendan J. Foran^1^ & Hanjong Paik^2,3^

^1^Microeletronics Technology Department, The Aerospace Corporation, El Segundo, California 90245, USA. ^2^School of Electrical and Computer Engineering, University of Oklahoma, Norman, Oklahoma 73019, USA. ^3^Center for Quantum Research and Technology, University of Oklahoma, Norman, Oklahoma 73019, USA. *Correspondence and requests for materials should be addressed to I.T.B. (e-mail: [intae.bae@aero.org](mailto:intae.%20bae@aero.org))

Supplementary Information


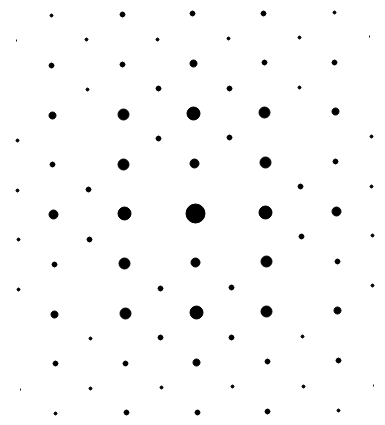

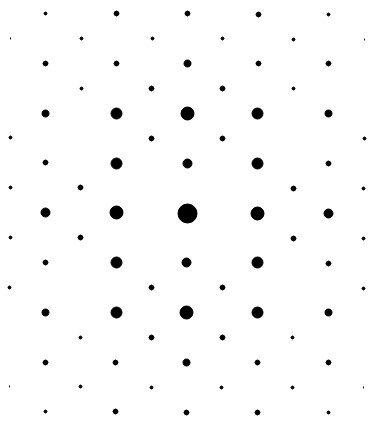

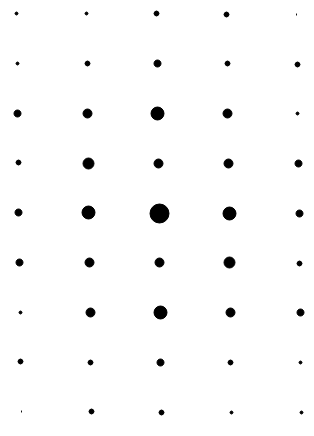

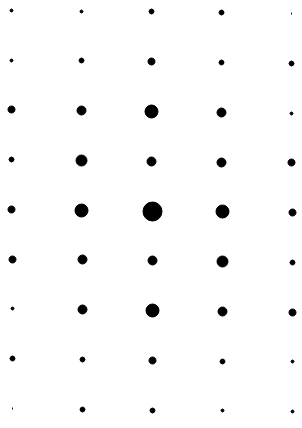


114_h_

**-**

**-**

**-**

**-**

213_h_

**-**

022_h_

**-**

222_h_

**-**

022_h_

**-**

110_h_

**-**

**-**

202_h_

**-**

**-**

213_h_

**-**

**-**

123_h_

**-**

**-**

112_h_

**-**

**-**

120_h_

**-**

113_h_

**-**

102_h_

**-**

104_h_

Supplementary Figure S1. Electron diffraction simulation of rhombohedral BFO^19^ along (a) [110] _h_ (a’), [$0\bar{1}0$]_h_, (b) [$\bar{1}11]$_h_, and (b’) [211]_h_ zone axes. Details on electron diffraction simulation can be found elsewhere^18,21-25^.

202_h_

102_h_

**-**

006_h_

**-**

**-**

222_h_

114_h_

006_h_

112_h_

(a)

(a’)

(b’)

(b)

Supplementary Figures


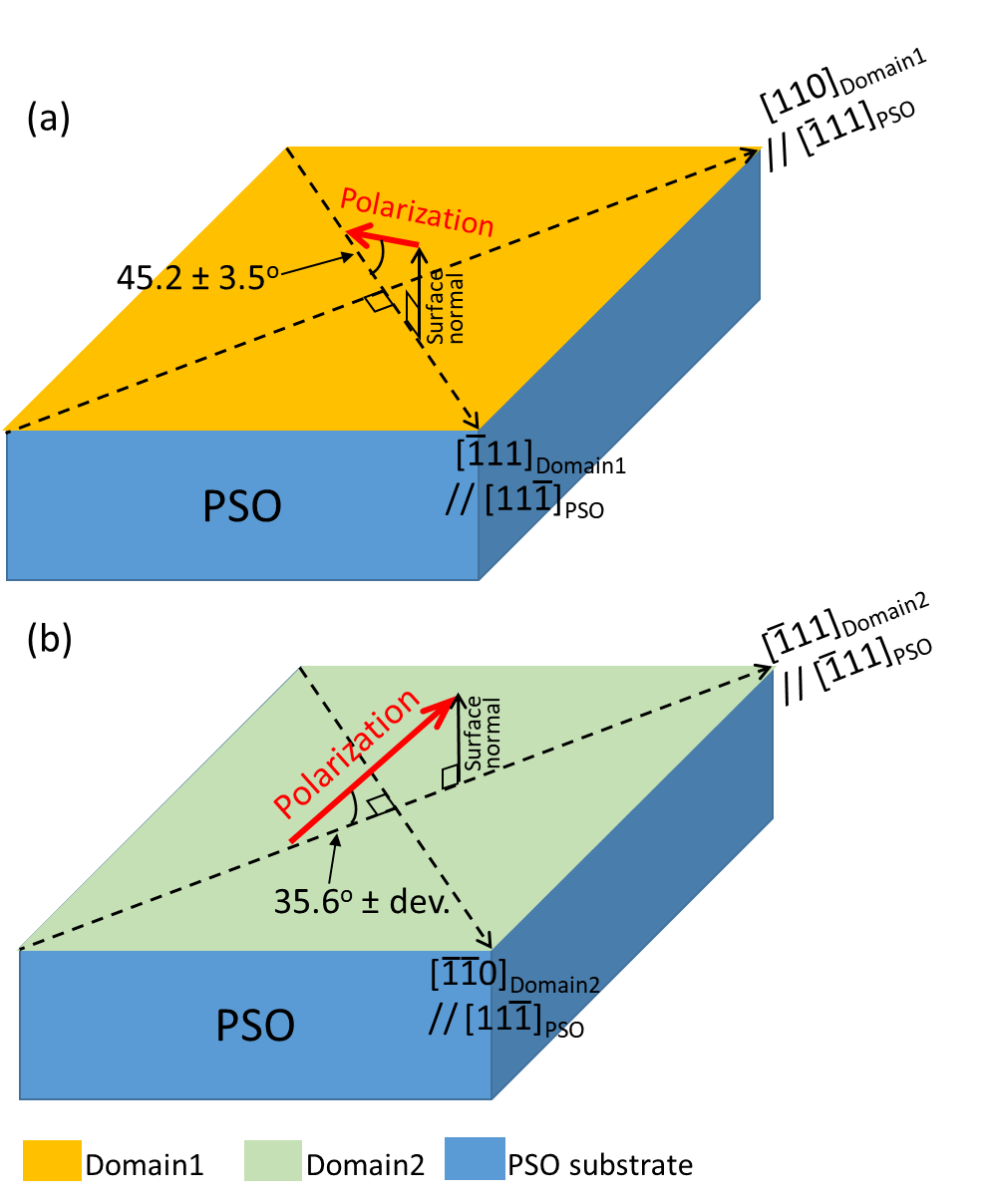


Supplementary Figure S2. Spontaneous polarization vectors of domains 1, (a), and 2, (b) are three dimensionally shown to show the relationship between polarization vectors and crystallographic orientations within each rhombohedral BFO domain. Note that while angle between polarization vector and BFO film surface in domain 1 is based on 4D-STEM measurement, that in domain 2 is assumed the sum of theoretical value of 35.6^o^ from unstrained rhombohedral BFO and polarization vector change (dev.) owing to the tensile strain caused by PSO substrate.

Supplementary Tables

| Hexagonal BFO | Pseudocubic BFO | Corresponding orthorhombic PSO orientation |
| --- | --- | --- |
| [241]_h_ (=[$\bar{4}\bar{2}1$]_h_) | [100]_pc_ | [010]_o_ |
| [211]_h_ (=[$\bar{1}11]$]_h_) | [110]_pc_ | $[\bar{1}11]$_o_ |
| $[0\bar{1}0$]_h_ (=[110]_h_) | [$\bar{1}10$]_pc_ | $[\bar{11}1]$_o_ |
| [001]_h_ | [111]_pc_ | [$\bar{1}10$]_o_ |
| $\left( 11\bar{3} \right)$_h_ | (0.5 -0.5 1.5)_pc_ | $\left( 211 \right)$_o_ |
| $\left( 2\bar{13} \right)$_h_ | (-0.5 0.5 1.5)_pc_ | ($1\bar{1}2$)_o_ |
| $\left( \bar{11}3 \right)$_h_ | (-0.5 0.5 -1.5)_pc_ | $\left( \bar{211} \right)$_o_ |
| $\left( \bar{2}13 \right)$_h_ | (0.5 -0.5 -1.5)_pc_ | ($\bar{1}1\bar{2}$)_o_ |
| ($1\bar{2}0$)_h_ | ($\bar{1}10$)_pc_ | $\left( \bar{12}1 \right)$_o_ |
| ($\bar{1}0\bar{4}$)_h,_ [=(1$\bar{1}\bar{4}$)_h_] | ($\bar{11}0$)_pc_ | $\left( 1\bar{21} \right)$_o_ |

Supplementary Table S1. Hexagonal notation conversion to pseudocubic notation. PSO orientations and planes corresponding to those of BFO are also shown.
